# Ternary ZnS/ZnO/Graphitic Carbon Nitride Heterojunction for Photocatalytic Hydrogen Production

**DOI:** 10.3390/ma17194877

**Published:** 2024-10-04

**Authors:** Asset Bolatov, Alida Manjovelo, Bilel Chouchene, Lavinia Balan, Thomas Gries, Ghouti Medjahdi, Bolat Uralbekov, Raphaël Schneider

**Affiliations:** 1 LRGP, CNRS, Université de Lorraine, F-54000 Nancy, France; assetbolatov@gmail.com (A.B.); alida.manjovelo8@etu.univ-lorraine.fr (A.M.); bilel.chouchene@univ-lorraine.fr (B.C.); 2 Center of Physical-Chemical Methods of Research and Analysis, Al-Farabi Kazakh National University, Al-Farabi Av., 71, Almaty 050040, Kazakhstan; bulat.ural@gmail.com; 3 CEMHTI-UPR 3079 CNRS, Site Haute Température, 1D Avenue de la Recherche Scientifique, F-45071 Orléans, France; lavinia.balan@cnrs-orleans.fr; 4 IJL, CNRS, Université de Lorraine, F-54000 Nancy, France; thomas.gries@univ-lorraine.fr (T.G.); ghouti.medjahdi@univ-lorraine.fr (G.M.)

**Keywords:** ZnS/ZnO/gCN heterojunction, photocatalytic hydrogen evolution, double Z-scheme mechanism

## Abstract

Ternary ZnS/ZnO/graphitic carbon nitride (gCN) photocatalysts were prepared by coupling gCN sheets with ZnO nanorods under solvothermal conditions followed by sulfurization using Na_2_S. SEM and TEM analyses show that small-sized ZnS particles (ca. 7.2 nm) deposit homogeneously on the surface of ZnO/gCN nanohybrids. Photoluminescence and electrochemical impedance spectroscopy show that ZnS allows for an enhanced charge separation efficiency as well as prolonged lifetime of photogenerated charge carriers, leading to improved hydrogen photoproduction under UV light irradiation compared to ZnO/gCN. Moreover, the deposition of ZnS nanoparticles improves the photostability of the ZnS/ZnO/gCN catalyst for hydrogen production. A double Z-scheme mechanism is proposed for hydrogen photoproduction using the ZnS/ZnO/gCN heterojunction.

## 1. Introduction

Green hydrogen (H_2_) energy is one of the most promising solutions to address the energy crisis and environmental pollution issue [1,2]. Indeed, H_2_ exhibits high specific energy density (120 kJ⋅g^−1^) and when H_2_ reacts with oxygen in an internal combustion engine or a fuel cell, the stored energy is released and only water is produced as a by-product [3]. Light-driven photocatalytic H_2_ production is a promising energy conversion technology allowing for the conversion of solar energy into carbon-free hydrogen [4,5]. In recent years, a large number of inorganic and organic photocatalysts like TiO_2_, ZnO, metal organic frameworks (MOFs) or graphitic carbon nitride (gCN) have been developed for water splitting [4,5,6,7]. However, the efficient conversion of light into the H_2_ energy vector remains challenging. The previously mentioned single-component photocatalysts suffer from a high recombination rate of photogenerated electron–hole pairs and from a lack of active sites on the surface of the catalyst to allow for efficient H_2_ production [4,5,6,7]. The cost of these catalysts as well as their large-scale practical applications also need to be considered as many of them require a co-catalyst, usually a noble metal like platinum, rhodium or ruthenium [8,9].

In recent years, the construction of heterojunctions between two or more semiconductors with suitable bandgap positions has been demonstrated to be of high value to improve the charge separation and transfer [7,10,11,12]. The built-in electric field produced at the interface of the semiconductors by their difference in potentials markedly improves the spatial separation of photogenerated charge carriers, thus inhibiting their recombination. Moreover, the use of a small bandgap semiconductor in the heterojunction allows for us to enhance the utilization of solar light, which is crucial for the development of photocatalytic hydrogen production technology [10,11,12,13]. The construction of heterojunctions between ZnO and gCN has recently been of great interest for various photocatalytic applications like pollutant degradation [14,15,16,17,18], NO oxidation [19], hydrogen peroxide [20] or H_2_ [15,16,17,19,21,22,23,24,25,26,27] production. These two semiconductors are cheap, easily prepared and their relative bandgap positions are well matched, making it easier to construct the heterojunction [28,29]. ZnO nanorods (NRs) are of high potential for photocatalytic applications due to their large specific surface area allowing them to absorb more light than spherical ZnO particles and thus generate more charge carriers [14]. More electrons and holes are also present on the catalyst surface due to the dimensional anisotropy of NRs, and their stability and electronic properties can easily be modelled based on the density functional theory (DFT) [30]. gCN is also of high interest for H_2_ photoproduction due to its bandgap of ca. 2.7 eV, allowing for its activation in the visible region until 460 nm and its energy band positions vs. NHE at pH = 7 of −1.3 V and +1.4 V vs. NHE for the conduction and the valence band, respectively, giving to gCN a high reducing potential for the conversion of water into H_2_ [31].

However, ZnO suffers from a modest stability and is easily photo-corroded by holes generated in the valence band during photocatalytic reactions, especially when irradiation is carried out over long periods [32]. A strategy to overcome this problem is to encapsulate the photocatalyst in another semiconductor with an adequate bandgap to form a ternary heterojunction. Due to its theoretical gap energy of about 3.7 eV and the positions of the valence and conduction bands located between those of ZnO and gCN, ZnS could meet this criterion. To our knowledge, only one report describes the preparation of the ZnO/ZnS/gCN heterojunction for H_2_ photoproduction [33]. This photocatalyst was prepared by loading ZnS onto ZnO followed by hybridization with gCN and exhibiting a H_2_ production rate of ca. 301 μmol h^−1^ g^−1^.

Herein, we report an alternative method for the preparation of ZnS/ZnO/gCN composite photocatalysts based on the sulfuration of preformed ZnO/gCN nanohybrids. Our results show that small-sized ZnS particles are evenly distributed at the surface of the ZnO/gCN catalyst, thus allowing to increase its photostability. Moreover, the ZnS/ZnO/gCN (20%) catalyst exhibits high activity and its H_2_ production rate is of 2548 μmol h^−1^ g^−1^, a value that favorably compares to the ZnO/gCN-based catalysts described in the literature.

## 2. Materials and Methods

### 2.1. Materials

Melamine (99%, Sigma Aldrich, Darmstadt, Germany), cyanuric chloride (99%, Acros Organics, Geel, Belgium), zinc acetate dihydrate Zn(OAc)_2_●2H_2_O (99+%, Fisher, Illkirch, France), sodium sulfide nonahydrate Na_2_S●9H_2_O (98%, Fischer), sodium sulfite Na_2_SO_3_ (98.5%, Fisher), tetrahydrofuran THF (99.85%, Fisher), sodium hydroxide (VWR, 98.5%) and anhydrous ethanol (Carlo Erba, Val de Reuil, France) were used as received.

### 2.2. Synthesis of gCN

Cyanuric chloride (2.5 g, 13.5 mmol) was added to melamine (2.5 g, 20 mmol) in 20 mL of a 1:1 THF/H_2_O mixture and the solution was stirred at room temperature for 5 h. The white gel obtained was dried at 60 °C for 12 h. The powder obtained was placed in a closed crucible and next heated for 1 h at 250 °C, then for 1 h at 350 °C and finally for 1 h at 450 °C with a heating rate of 10 °C.min^−1^ in a muffle furnace to prepare bulk gCN. After cooling at room temperature, gCN was obtained as a yellow powder and used without any purification.

### 2.3. Synthesis of ZnO/gCN Photocatalysts

ZnO NRs were prepared by the solvothermal method. Briefly, Zn(OAc)_2_ (2.33 mmol) was dissolved in 35 mL of absolute ethanol. Separately, NaOH (11.65 mmol) was dissolved in 35 mL of absolute ethanol and then added to the Zn(OAc)_2_ solution dropwise. The mixture was magnetically stirred for 30 min at room temperature, then transferred into a Teflon flask in an autoclave and heated for 24 h at 160 °C. After cooling to room temperature, the dispersion was centrifuged (4000 rpm for 20 min) and the white solid obtained was washed with distilled water (3 × 20 mL) and absolute ethanol (3 × 20 mL), then dried at 70 °C overnight. The mass of ZnO NRs obtained was ca. 150 mg.

The ZnO/gCN(20%) catalyst was prepared using the same protocol except that 30 mg of gCN was added to the mixture after 30 min of stirring at room temperature followed by sonication for 20 min before transfer in the autoclave.

### 2.4. Sulfuration of ZnO/gCN. Preparation of ZnS/ZnO/gCN Catalysts

The preparation of ZnS/ZnO/gCN (20%) was carried out by sulfuration of the ZnO/gCN (20%) catalyst as previously described [34]. Briefly, Na_2_S (480 mg) was dissolved in 50 mL of water and 120 mg of the ZnO/gCN (20%) catalyst was added. The mixture was magnetically stirred at 70 °C for 10 h. The powder was recovered by centrifugation (4000 rpm for 15 min), washed with water (3 × 20 mL) and absolute ethanol (3 × 20 mL) and dried overnight at 70 °C before use.

### 2.5. Photocatalytic Hydrogen Production

Photocatalytic H_2_ production tests were performed at 25 °C in a sealed quartz reactor using as light source a 300 W full spectrum Xenon lamp (Asahi Spectra, Torrance, CA, USA) equipped with a 250–385 nm filter limiting the irradiation wavelengths to the UV range. Typically, 100 mg of photocatalyst was dispersed in 100 mL of an aqueous solution containing Na_2_S (0.4 M)/Na_2_SO_3_ (0.3 M) as sacrificial substrates. Before irradiation, the reactor was purged with N_2_ for 1 h to remove O_2_. The amount of H_2_ produced was measured each hour using a gas chromatograph (GC) equipped with a thermal conductivity detector (TCD) and high-purity argon as the carrier gas. The reaction mixture was continuously stirred during the experiment to ensure the appropriate dispersion of the photocatalyst.

### 2.6. Characterizations

Powder X-ray diffraction (XRD) was used to investigate the crystallinity and the phase of the photocatalysts using an X-ray diffractometer (Panalytical X’Pert pro MPD diffractometer, Malvern, Orsay, France, with Cu Kα radiation = 0.15406 nm). The morphology and the microstructure of the photocatalysts were examined by transmission electron microscopy (TEM) using Philips CM200 equipment (Philips, Suresnes, France) operating at an acceleration voltage of 200 kV equipped with energy-dispersive X-ray (EDX) and by scanning electron microscopy (SEM, JEOL JSM-6490 LV and JEOL JSM IT800 instruments, Croissy, France). The surface composition and the chemical state of elements were characterized by X-ray photoelectron spectroscopy (XPS) using a Gammadata Scienta SES 200-2 spectrometer (Uppsala, Sweden). The functional groups present in the catalysts were examined using Fourier transform infrared (FT-IR) spectroscopy using a Brucker ALPHA II FT-IR spectrometer (Bruker, Palaiseau, France).

UV–visible diffuse reflectance spectra (DRS) were recorded on a Shimadzu 2600–2700 spectrometer (Shimadzu, Le Luzard, France). Photoluminescence (PL) spectra were recorded on a Horiba Fluoromax spectrofluorometer (Horiba Jobin Yvon, Longjumeau, France) equipped with a Xenon lamp as the excitation source.

The electrochemical impedance spectroscopy (EIS) response of the samples was measured from 500 kHz to 10 mHz for low amplitudes of ±10 mV at open circuit potential (OCP) under dark conditions using a potentiostat BioLogic SP150 in a conventional three-electrode cell. The photocatalysts were coated onto FTO glass for the working electrode. Platinum (Pt) coil was used as the counter electrode and Ag/AgCl as the reference electrode in a 0.1 M Na_2_SO_4_ aqueous solution.

## 3. Results

### 3.1. Photocatalyst Synthesis and Characterization

**Scheme 1:**
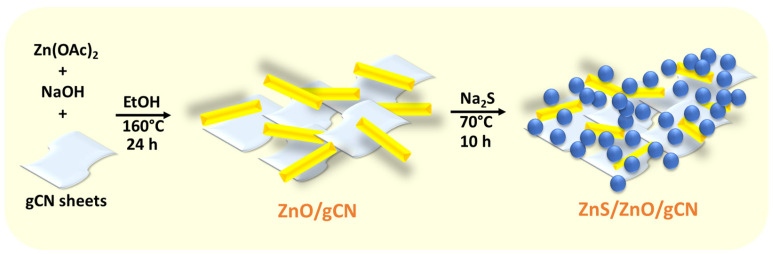
Schematic illustration of ZnO/gCN and ZnS/ZnO/gCN photocatalyst synthesis.

The ZnO/gCN photocatalysts were prepared via a solvothermal method in which Zn(OAc)_2_ is hydrolyzed into ZnO clusters that grow at 160 °C into NRs that associate with gCN sheets (Scheme 1). The amount of gCN was varied during synthesis in order to prepare photocatalysts containing 10, 20 or 30 wt% gCN relative to ZnO. The ZnO/gCN catalyst sulfuration was conducted at 70 °C using an aqueous solution of Na_2_S. During this step, Zn^2+^ cations are detached from the surface of ZnO and associate with S^2−^ to form ZnS particles that deposit on the surface of the ZnO/gCN composite to form the ternary ZnS/ZnO/gCN catalyst.

**Figure 1:**
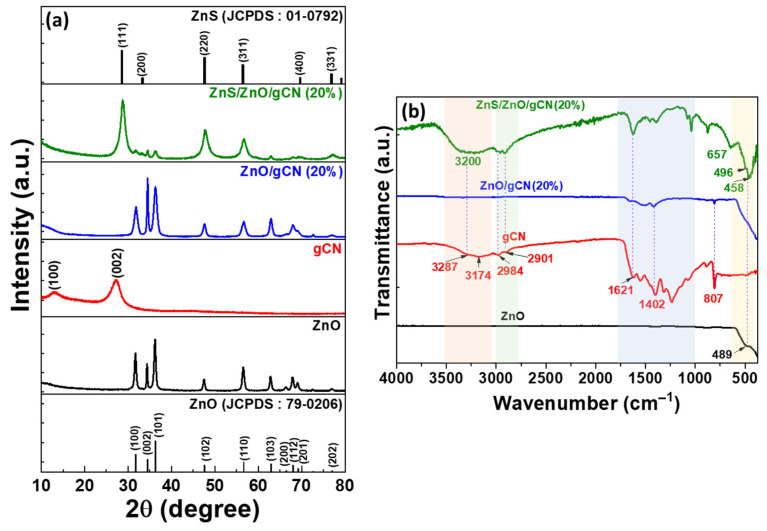
(**a**) XRD patterns and (**b**) FT-IR spectra of ZnO, ZnO/gCN (20%) and ZnS/ZnO/gCN (20%) catalysts.

X-ray diffraction (XRD) analyses were conducted to determine the phase structure of the catalysts, and the results are shown in Figure 1a. Pure ZnO shows signals at 2ϴ values of 31.66, 34.33, 36.22, 47.48, 56.54, 62.84, 66.37, 67.87, 69.13 and 76.86°, corresponding to the hexagonal wurtzite structure of ZnO (JCPDS No 79-0206) [14]. For gCN, the two signals observed at 13.08 and 27.32° can be assigned to the (100) in-planar structural packing of tri-s-triazine moieties and (002) interlayer graphitic stacking of aromatic rings [14]. The XRD signals of gCN can only weakly be observed in the ZnO/gCN composite, suggesting that gCN was partially delaminated during the solvothermal synthesis used for the synthesis of the composite. It is noteworthy that this decrease in the number of gCN polymeric layers should be beneficial for the separation of charge carriers [35]. The XRD signals of ZnO and gCN in the ZnS/ZnO/gCN (20%) composite are significantly weaker than those of the ZnO/gCN (20%) nanohybrid, which confirms the covering of ZnO/gCN (20%) by ZnS. The broad peaks observed at 28.82, 47.72 and 56.85° correspond to the (111), (200) and (220) planes of the cubic sphalerite phase of ZnS (JCPDS No 76-0704). No impurities were detected, thus indicating the purity of the photocatalysts prepared. The XRD results obtained for ZnO, ZnO/gCN and ZnS/ZnO/gCN catalysts, refined using the Rietveld method, confirm the hexagonal and cubic structure of ZnO and ZnS, respectively (Figure S1).

The functional groups present in the photocatalysts were further investigated by FT-IR (Figure 1b). For pure ZnO, the vibrational mode of Zn-O bonds is observed at 489 cm^−1^. The FT-IR spectrum of gCN shows signals between 3287 and 2901 cm^−1^ corresponding to uncondensed N-H and NH_2_ bonds and to chemisorbed water molecules. The peaks observed between 1621 and 1250 cm^−1^ can be assigned to the stretching vibrations of C=N and C-N functions in aromatic heterocycles, while the sharp peak at 807 cm^−1^ can be attributed to the tri-s-triazine ring-breathing vibration [14]. The FT-IR spectrum of ZnO/gCN (20%) composite exhibits the characteristic peaks of both ZnO and gCN, confirming that these two semiconductors were well combined during the solvothermal synthesis. After ZnS is deposited, many signals, especially those of gCN, are hidden. The FT-IR spectrum of the ZnS/ZnO/gCN (20%) composite particularly exhibits a broad signal centered at ca. 3200 cm^−1^ corresponding to adsorbed water molecules, while the peaks observed at 657, 496 and 458 cm^−1^ are likely associated with asymmetric Zn-S stretching, Zn-O stretching and symmetric Zn-S stretching, respectively [36].

**Figure 2:**
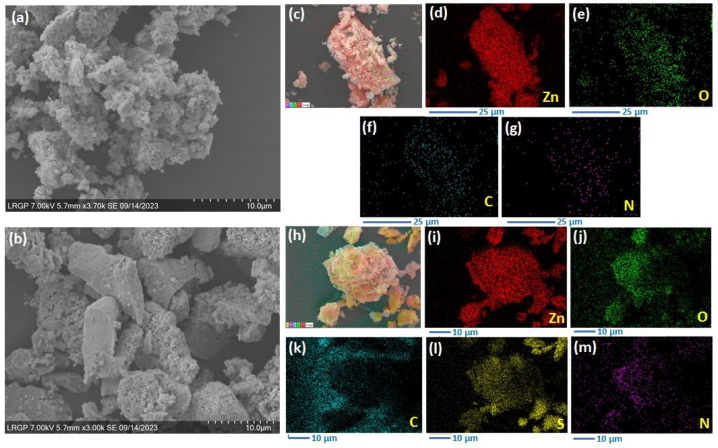
SEM images of (**a**) ZnO/gCN (20%) and (**b**) ZnS/ZnO/gCN (20%) catalysts; (**c**–**m**) EDX elemental mappings of the composite photocatalysts.

The morphology and composition of ZnO/gCN (20%) and ZnS/ZnO/gCN (20%) catalysts were first studied by scanning electron microscopy (SEM) (Figure 2). As can be seen in Figure 2a, small ZnO NRs are distributed on the surface of gCN sheets. The size of ZnO/gCN (20%) assemblies varies from a few hundred nanometers to several micrometers. The SEM-EDX-associated elemental mapping shows the presence of Zn, O, C and N elements, further confirming that the ZnO/gCN composite was successfully prepared (Figure 2c–g). The ZnS/ZnO/gCN (20%) catalyst has a denser structure, suggesting that ZnS particles are deposited on the surface and in the pores of the ZnO/gCN hybrid (Figure 2b). In the core of the micrometric particles produced, ZnO NRs can be observed. The related elemental mapping shows Zn, O, C, S and N elements, indicating that the ternary composite photocatalyst was synthesized (Figure 2h–m).

**Figure 3:**
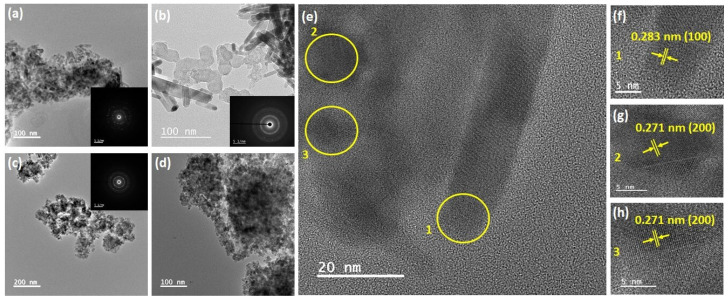
TEM images of (**a**) ZnO NRs, (**b**) ZnO/gCN (20%), (**c**,**d**) ZnS/ZnO/gCN (20%) photocatalysts (the insets of (**a**–**c**) correspond to SAED patterns). (**e**–**h**) HR-TEM images of the ZnS/ZnO/gCN (20%) heterostructure.

The morphology and crystallinity of the photocatalysts were further investigated by transmission electron microscopy (TEM) and HR-TEM. Pure ZnO NRs have an average length of 55 nm, a diameter of ca. 12 nm and their surface is smooth (Figure 3a). After association with gCN by hydrothermal treatment, ZnO NRs were found to be randomly distributed on the surface of gCN sheets (Figure 3b). As previously observed by SEM, the ZnS/ZnO/gCN (20%) heterostructures become much denser after ZnS deposition and ZnS nanoparticles with an average diameter of ca. 7.2 nm, and were found to be homogeneously distributed on ZnO NRs and gCN sheets (Figure 3c,d). The selected area of electron diffraction patterns associated with TEM images further confirm the high crystallinity of the samples (inset of Figure 3a–c). Figure 3e is an HR-TEM image of the ternary photocatalyst, which confirms that ZnS, ZnO and gCN are strongly bound. The measured lattice spacing of 0.283 and 0.271 nm correspond to (100) and (200) planes of hexagonal ZnO and cubic ZnS, respectively, which agrees with the XRD results. TEM images demonstrate that ZnS, ZnO and gCN are well in contact, which should allow photogenerated charge carriers to move across the heterostructured catalyst.

**Figure 4:**
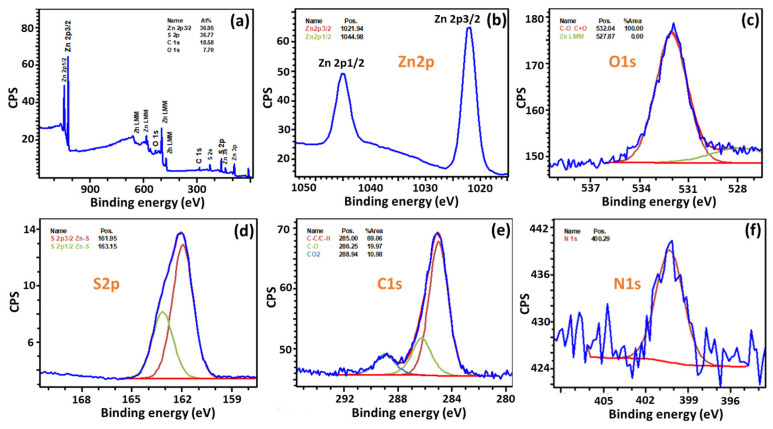
(**a**) Overview XPS spectrum of the ZnS/ZnO/gCN (20%) photocatalyst. HR-XPS spectra of (**b**) Zn 2p, (**c**) O 1s, (**d**) S 2p, (**e**) C 1s and (**f**) N 1s.

X-ray photoelectron spectroscopy (XPS) was performed to analyze the chemical composition and the oxidation state of elements composing ZnO/gCN (20%) and ZnS/ZnO/gCN (20%) catalysts. The overview XPS spectrum of ZnO/gCN (20%) and the HR-XPS spectra of Zn 2p, O 1s, C 1s and N 1s are given in Figure S2 and are characteristic of this nanohybrid. Two signals can be observed for Zn 2p at 1021.17 and 1044.26 eV assigned to Zn 2p_3/2_ and Zn 2p_1/2_, respectively, and correspond to Zn(+2) in the ZnO lattice [37]. For C 1s, the major component at 285 eV can be assigned to C-C bonds like graphite and amorphous carbon while the signal at 288.40 eV is associated with carbon atoms linked to two N atoms (N-C=N) [38]. The N 1s peak can be deconvoluted into three signals, 398.70, 400.22 and 401.25 eV, which can be assigned to N atoms bonded to two C atoms (C=N-C), N linked to three C atoms (N-(C)_3_), and H-N-C bonds, respectively [39]. The overview XPS spectrum of ZnS/ZnO/gCN (20%) is provided in Figure 4a and shows only the presence of Zn, O, S, C and N elements, indicating the successful engineering of the ternary photocatalyst. The HR-XPS spectrum shows the typical signals of Zn(+2) at 1021.94 and 1044.98 eV that can be assigned to Zn 2p_3/2_ and Zn 2p_1/2_, respectively (Figure 4b). The shift observed compared to ZnO/gCN confirms the sulfuration of ZnO. For O 1s, only one signal corresponding to adsorbed oxygen species can be observed at 532.04 eV, while O associated with Zn-O bonds was not detected (Figure 4c) [40]. This result confirms that ZnO is not present on the surface of the ZnS/ZnO/gCN composite and that the catalyst structure is close to core/shell ZnO-gCN/ZnS. The S 2p signal can be deconvoluted into two signals located at 161.95 and 163.15 eV, corresponding to S 2p_3/2_ and S 2p_1/2_, respectively, which agrees well with values reported for ZnS (Figure 4d) [41]. The C 1s spectrum shows three peaks located at 285.0, 286.25 and 288.94 eV that can be assigned to sp^2^ hybridized C=C bonds, and to C-O and CO_2_ bonds, respectively (Figure 4e). A weak signal can also be observed for N 1s at 400.29 eV (Figure 4f). However, the characteristic peaks of C 1s and N 1s present in gCN could not be detected, further confirming that gCN is only present in the core of the catalyst.

**Figure 5:**
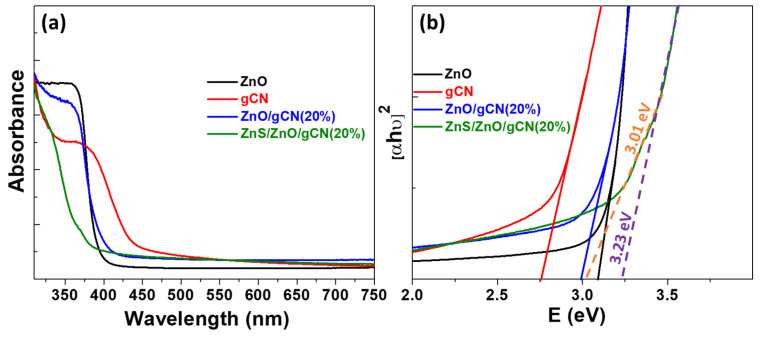
(**a**) UV–visible diffuse reflectance spectra of ZnO, gCN, ZnO/gCN (20%) and ZnS/ZnO/gCN (20%) photocatalysts. (**b**) The corresponding Tauc plots used to determine the bandgap.

UV–visible diffuse reflectance spectroscopy (DRS) was used to investigate the optical absorption properties of the photocatalysts (Figure 5a). The energy bandgaps *Eg* were determined using the Tauc plots obtained via the formula (*αhγ*)^2^ = A (*hγ* − *Eg*), where *α*, *hγ* and A are the absorption coefficient, the photon energy and a proportionality constant, respectively (Figure 5b). The absorption edge of ZnO NRs is located at ca. 400 nm and its bandgap is of 3.09 eV, which agrees well with literature [14]. Pure gCN absorbs in the visible region (absorption edge at ca. 490 nm) due to π-π* and n-π* transitions of the triazine units [42], and its bandgap is 2.75 eV. After associating with gCN and ZnO, the energy bandgap of the composite (2.99 eV) was found to be lower than that of ZnO, indicating that the visible light absorption of the ZnO/gCN (20%) hybrid is enhanced compared to ZnO, which is favorable for photocatalytic reactions. The ZnS/ZnO/gCN (20%) ternary composite obtained after ZnS deposition shows not only a very high absorption in the UV but also a slightly enhanced absorption in the visible region, indicating that more photons could be absorbed between 400 and 800 nm. The visible absorption band tail likely originates from the enhanced light scattering induced by the ZnS deposition [43,44]. The bandgap of ZnS in the ZnS/ZnO/gCN catalyst is 3.23 eV; this value is in accordance with previous reports [33]. The second contribution observed at 3.01 eV corresponds to the ZnO/gCN (20%) composite.

### 3.2. Photocatalytic Performance and Stability

In the first set of experiments, the photocatalytic H_2_ evolution performance of gCN, ZnO and ZnO/gCN materials was evaluated under UV light irradiation by adding a sacrificial substrate in the aqueous solution but without any co-catalyst. Among the various sacrificial substrates evaluated (Na_2_S/Na_2_SO_3_, methanol, glycerol, triethanolamine), the best results were obtained using Na_2_S (0.4 M)/Na_2_SO_3_ (0.3 M), and these compounds were used in the study.

**Figure 6:**
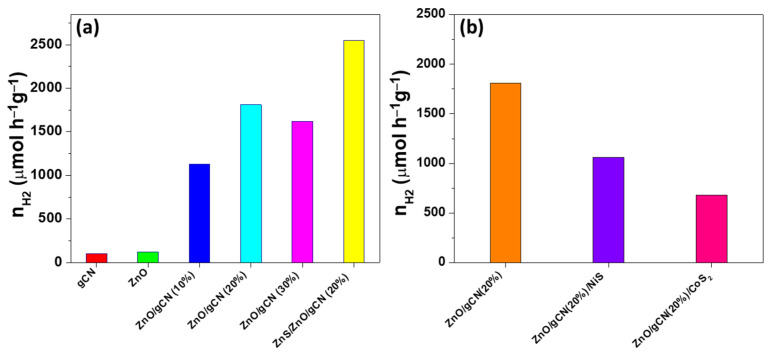
(**a**) Hydrogen production rates of gCN, ZnO and ZnO/gCN (10, 20 or 30%) photocatalysts. (**b**) Influence of NiS and CoS_2_ cocatalysts on the H_2_ production rate.

Despite the adequate positions of gCN and ZnO conduction bands relative to the H^+^/H_2_ couple, these materials exhibit modest activity for H_2_ photoproduction (105 and 126 μmol h^−1^ g^−1^, respectively) (Figure 6a). The association of ZnO with gCN markedly increases the H_2_ production rate with a maximum H_2_ production rate of 1813 μmol h^−1^ g^−1^ for the heterojunction containing 20 wt% relative to ZnO. The photocatalytic activity decreases when increasing the gCN loading to 30 wt%, suggesting that a loading that is too high in gCN favors the charge carrier recombination. In subsequent experiments, the loading of 20 wt% gCN relative to ZnO will be maintained.

In order to improve the separation of the charge carriers and thus the H_2_ photoproduction, the association of the ZnO/gCN catalyst with co-catalysts was also evaluated. The NiS and CoS_2_ co-catalysts were chosen because of their low cost compared to a noble metal like Pt, but also due to their effectiveness when combined with gCN or ZnO [45,46]. The co-catalysts were photo-deposited on the surface of the ZnO/gCN (20%) composite as previously described [45,46]. However, a decrease in activity was observed, likely due to blocking of the photocatalyst active sites after deposition of the co-catalysts (Figure 6b).

**Figure 7:**
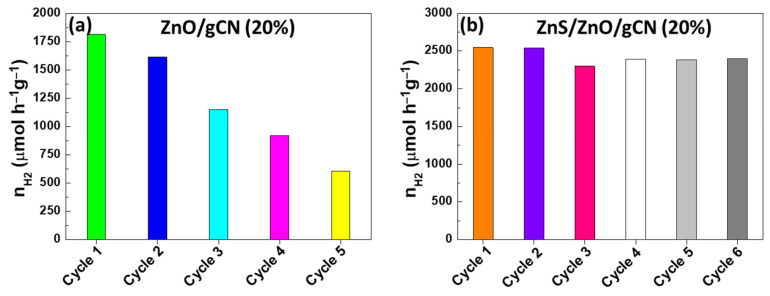
Cycle performance of (**a**) ZnO/gCN (20%) and (**b**) ZnS/ZnO/gCN (20%) photocatalysts.

The ZnO/gCN (20%) catalyst was found to be of modest stability during photocatalytic experiments, and a constant decline in the H_2_ production rate from 1813 to 604 μmol h^−1^ g^−1^ is observed after five cycles (Figure 7a). Under similar reaction conditions, the ZnS/ZnO/gCN (20%) catalyst exhibits significantly increased activity compared to ZnO/gCN (20%) with a H_2_ production rate of 2548 μmol h^−1^ g^−1^ in the first cycle, indicating that ZnS improves the charge transfer rate as well as the UV light utilization efficiency (Figure 6a and Figure 7b). Moreover, the photocatalytic performance of the ZnS/ZnO/gCN (20%) catalyst only weakly decreases during repeated cycles (from 2548 to 2394 μmol h^−1^ g^−1^ after six cycles), which confirms that embedding ZnO/gCN (20%) into ZnS increases the photocatalyst stability. As can be seen from Table 1, the H_2_ production rate of the ternary ZnS/ZnO/gCN (20%) catalyst is markedly higher than that of most of the binary ZnO/gCN catalysts reported to date. The significantly improved photocatalytic activity of the ZnS/ZnO/gCN (20%) catalyst likely originates from its unique structure in which ZnO and gCN are protected from any photocorrosion by ZnS.

### 3.3. Photocatalytic Mechanism

**Figure 8:**
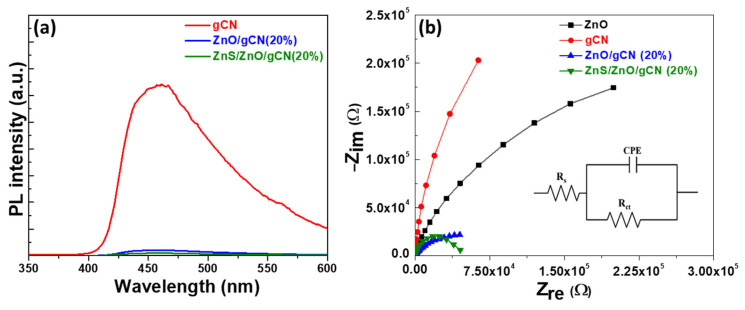
(**a**) PL emission spectra of gCN and ZnO/gCN (20%) photocatalysts; (**b**) EIS Nyquist plots for ZnO, gCN, ZnO/gCN (20%) and ZnS/ZnO/gCN (20%) photocatalysts. The inset of (**b**) is the Randles equivalent circuit used for fitting impedance spectra (Rs is the solution resistance, R_ct_ is the charge transfer resistance and CPE is the constant phase element).

The separation and the recombination of photogenerated charge carriers were first investigated by photoluminescence (PL) spectroscopy. After excitation at 380 nm, gCN shows a strong blue PL centered at ca. 458 nm, which mainly originates from n-π* transitions associated with the lone pairs of electrons present on N atoms in the gCN framework (Figure 8a) [47]. After association of gCN with ZnO, the PL intensity markedly decreases, indicating that the recombination of electron–hole pairs is hindered and that the charge separation is enhanced. The charge transfer is further improved in the ZnS/ZnO/gCN (20%) photocatalyst as almost no PL is observed.

Electrochemical impedance spectroscopy (EIS) measurements were also conducted in the dark to further study the charge transfer rate. As shown in Figure 8b, the arc radius of the ZnS/ZnO/gCN (20%) composite is smaller than that of ZnO, gCN and ZnO/gCN (20%) materials, indicating an enhanced electronic conductivity in the ground state, which agrees with the above-described PL measurements. The charge transfer resistance R_CT_ was determined using the Randles equivalent model (see Table S1 for R_s_ and R_ct_ values, CPE parameters Q and α, and inset of Figure 8b).

The energy band structure and the positions of the maximum valence band (VB) and the minimum conduction band (CB) of ZnO, gCN and ZnS versus the normal hydrogen electrode potential (NHE) were calculated using Equations (1) and (2) based on the Mulliken electronegativity theory: [48]

*E*_VB_ = χ − *E*^e^ + 0.5 *E*g (1)

*E*_CB_ = *E*_VB_ − *E*g (2)

where *E*_VB_ is the VB potential and *E*_CB_ the CB potential, χ is the absolute electronegativity of the semiconductor (5.89, 4.42 and 5.26 for ZnO, gCN and ZnS, respectively), *E*g is the bandgap of the semiconductor determined from UV–visible DRS, and *E*^e^ is the energy of free electrons on the hydrogen scale (ca. 4.5 eV).

**Figure 9:**
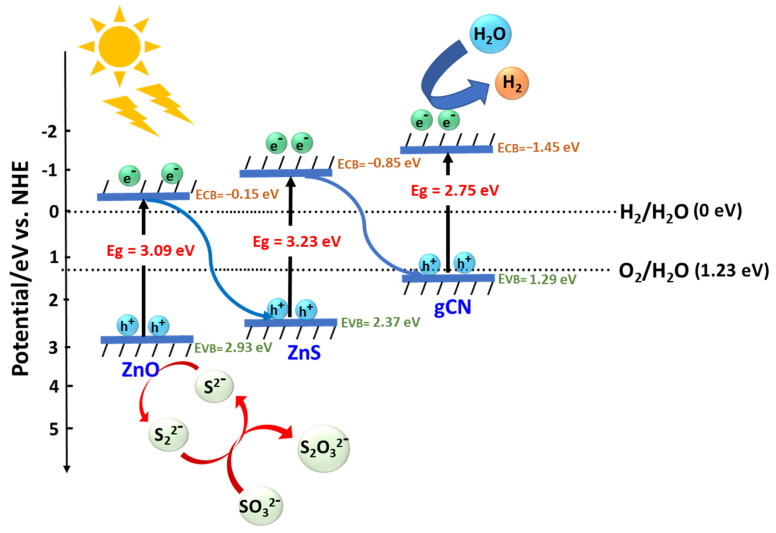
Double Z-scheme mechanism for the ZnS/ZnO/gCN (20%) photocatalyst under UV light irradiation.

Water reduction could be achieved by the successive transfer of photogenerated electrons from gCN to ZnS and then to ZnO (type II mechanism), but the potential difference between the conduction band of ZnO (−0.33 eV) and the H_2_/H_2_O couple is small (0.33 eV) and would not allow for an effective reduction of water into H_2_. For the ZnS/ZnO/gCN heterojunction, a double Z-scheme transfer pathway is privileged over a type II mechanism (Figure 9), which agrees with the literature [17,21,33]. Under UV light irradiation, electrons in the valence bands of ZnO, ZnS and gCN are promoted to the respective conduction bands, while holes are generated in the valence bands. Electrons in the conduction band of ZnO combine with the holes in the valence band of ZnS. Simultaneously, electrons in the conduction band of ZnS combine with the holes in the valence band of gCN. The electrons thus accumulated in the conduction band of gCN exhibit the highest reducing power to convert H_2_O molecules into H_2_. At the other end of the catalytic chain, holes in the valence band of ZnO are consumed by the Na_2_S/Na_2_SO_3_ sacrificial agents.

## 4. Conclusions

In summary, heterostructured photocatalysts associating with ZnS, ZnO and gCN were efficiently prepared by sulfurization of ZnO/gCN nanohybrids. ZnS nanoparticles thus produced are homogeneously distributed on the surface of ZnO/gCN and play a key role in the inhibition of the ZnO/gCN photocorrosion under UV light irradiation. An improved spatial separation of photogenerated charge carriers, as well as of their lifetime, was also demonstrated for the ZnS/ZnO/gCN photocatalyst. A double Z-scheme mechanism accounts for the efficient photocatalytic hydrogen evolution using the ZnS/ZnO/gCN (20%) catalyst.

## Figures and Tables

**Table 1:** Comparisons of the performance of catalysts combining ZnO with gCN for hydrogen photoproduction.

Catalyst	Light Source	Sacrificial Substrate	Co-Catalyst	H_2_ Production Rate (μmol h^−1^ g^−1^) or H_2_ Amount (μmol)	Reference
gCN/O-defective ZnO	300 W Xe lamp with UV filter	TEOA ^a^	-	322 μmol h^−1^ g^−1^	[17]
gCN/N-doped ZnO	300 W Xe lamp	MeOH	-	152.7 μmol h^−1^ g^−1^	[21]
gCN/ZnO NRs arrays	Simulated solar light	Na_2_S/Na_2_SO_3_	-	33 μmol after 3 h	[22]
gCN/Sm-doped ZnO	300 W Xe lamp with UV filter	Lactic acid	-	10,250 μmol h^−1^ g^−1^	[23]
gCN/N-doped ZnO	300 W Xe lamp with UV filter	TEOA	Pt (1.5 wt%)	780 μmol h^−1^ g^−1^	[19]
gCN/ZnO	300 W Xe lamp with UV filter	MeOH	-	70 μmol after 1 h	[24]
B-doped gCN/ZnO	300 W Xe lamp	TEOA	Pt (2 wt%)	357 μmol h^−1^ g^−1^	[25]
B-doped gCN/C,N-doped ZnO	125 W Xe lamp	MeOH	-	7020 μmol h^−1^ g^−1^	[15]
gCN/ZnO + eosin Y as sensitizer	400 W Xe lamp	TEOA	-	1358 μmol h^−1^ g^−1^	[16]
gCN/Ni-doped ZnO/C microspheres	300 W Xe lamp	TEOA	Pt (2 wt%)	336 μmol h^−1^ g^−1^	[26]
gCN/ZnO/ZnS	300 W Xe lamp	Na_2_S/Na_2_SO_3_	-	301 μmol h^−1^ g^−1^	[32]
gCN/ZnO/ZnS	300 W Xe lamp	Na_2_S/Na_2_SO_3_	-	2548 μmol h^−1^ g^−1^	This work

^a^ TEOA: triethanolamine.

## Data Availability

The original contributions presented in the study are included in the article/Supplementary Material, further inquiries can be directed to the corresponding author.

## Supplementary Materials

The following supporting information can be downloaded at https://www.mdpi.com/article/10.3390/ma17194877/s1. Figure S1. Rietveld refinement result of the powder XRD data for (a) ZnO, (b) ZnO/gCN and (c) ZnS/ZnO/gCN. The green curve illustrates the difference between data (blue curve) and simulation (red curve). Figure S2: (a) Overview XPS spectrum of the ZnO/gCN (20%) photocatalyst. (b–e) are the HR-XPS spectra of Zn 2p, O 1s, C 1s and N 1s elements, respectively. Table S1. Impedance parameters obtained after fitting the EIS curves with the Randles equivalent model.
