# Supplementary material for: Ternary ZnS/ZnO/Graphitic Carbon Nitride Heterojunction for Photocatalytic Hydrogen Production
Source: Materials (Basel). 2024 Oct 4;17(19):4877. doi: 10.3390/ma17194877 (PMC11477571; doi:10.3390/ma17194877)
Supplement: Supplementary file 1 [file materials-17-04877-s001.zip › materials-3219793-supplementary.pdf]

SUPPORTING INFORMATION

Ternary ZnS/ZnO/Graphitic Carbon Nitride Heterojunction for Photocatalytic Hydrogen Production

Asset Bolatov [1,2], Alida Manjovelo [1], Bilel Chouchene [1], Lavinia Balan [3], Thomas Gries [4], Ghouti Medjahdi [4], Bolat Uralbekov [2] and Raphaël Schneider [1,*]

[1] LRGP, CNRS, Université de Lorraine, F-54000 Nancy, France
[2] Center of Physical-Chemical Methods of Research and Analysis, Al-Farabi Kazakh National University, Al-Farabi Av., 71, Almaty 050040, Kazakhstan
[3] CEMHTI-UPR 3079 CNRS, Site Haute Température, 1D Avenue de la Recherche Scientifique, F-45071 Orléans, France
[4] IJL, CNRS, Université de Lorraine, F-54000 Nancy, France
[*] Correspondence: raphael.schneider@univ-lorraine.fr; Tel.: +33-3-72743790

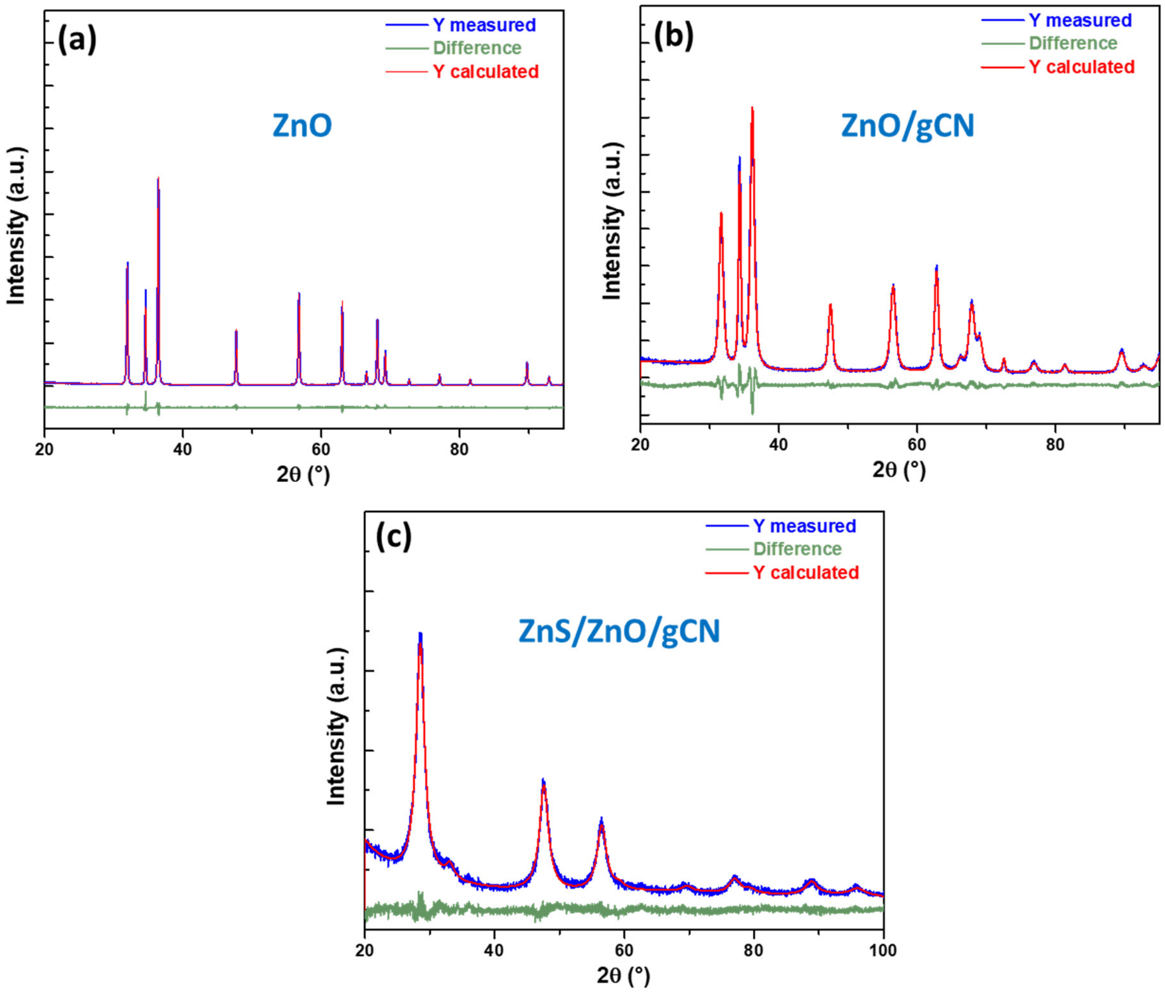

Figure S1. Rietveld refinement result of the powder XRD data for (a) ZnO, (b) ZnO/gCN and (c) ZnS/ZnO/gCN. The green curve illustrates the difference between data (blue curve) and simulation (red curve).

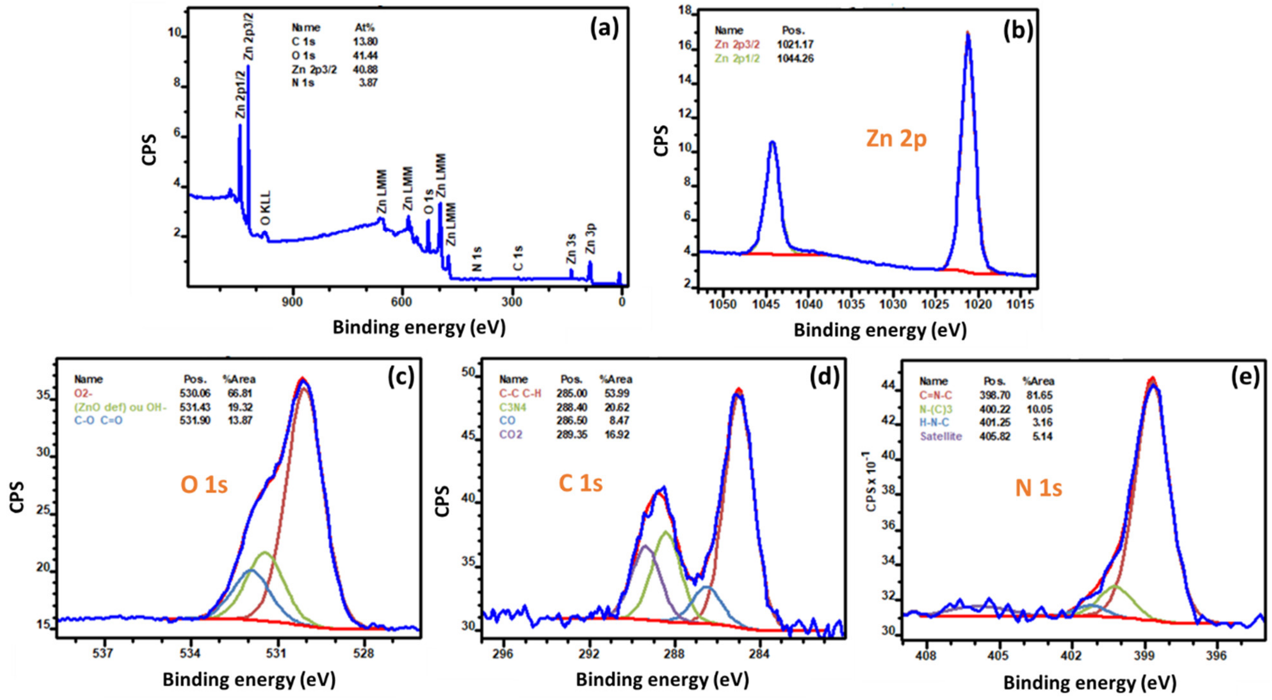

Figure S2. (a) Overview XPS spectrum of the ZnO/gCN (20%) photocatalyst. (b-e) are the HR-XPS spectra of Zn 2p, O 1s, C 1s and N 1s elements, respectively.

Table S1. Impedance parameters obtained after fitting the EIS curves with the Randles equivalent model.

Sample	Rs (Ω)	Q ($F.s^{\alpha-1}$)	α	Rct (Ω)
gCN	72.83	$6{,}912.10^{-6}$	0.9519	946489
ZnO	24.81	$4{,}859.10^{-6}$	0.7396	560225
ZnO/gCN	56.57	$20{,}94.10^{-6}$	0.5917	83745
ZnS/ZnO/gCN	234.2	$13{,}21.10^{-6}$	0.955	43651
